# Communication Networks in the Brain: Neurons, Receptors, Neurotransmitters, and Alcohol

**Published:** 2008

**Authors:** David M. Lovinger

**Keywords:** Alcohol and other drug effects and consequences, brain, neurons, neuronal signaling, synaptic transmission, neurotransmitter receptors, neurotrophins, steroid hormones, γ-aminobutyric acid (GABA), glutamate, dopamine, adenosine, serotonin, opioids, endocannabinoids

## Abstract

Nerve cells (i.e., neurons) communicate via a combination of electrical and chemical signals. Within the neuron, electrical signals driven by charged particles allow rapid conduction from one end of the cell to the other. Communication between neurons occurs at tiny gaps called synapses, where specialized parts of the two cells (i.e., the presynaptic and postsynaptic neurons) come within nanometers of one another to allow for chemical transmission. The presynaptic neuron releases a chemical (i.e., a neurotransmitter) that is received by the postsynaptic neuron’s specialized proteins called neurotransmitter receptors. The neurotransmitter molecules bind to the receptor proteins and alter postsynaptic neuronal function. Two types of neurotransmitter receptors exist—ligand-gated ion channels, which permit rapid ion flow directly across the outer cell membrane, and G-protein–coupled receptors, which set into motion chemical signaling events within the cell. Hundreds of molecules are known to act as neurotransmitters in the brain. Neuronal development and function also are affected by peptides known as neurotrophins and by steroid hormones. This article reviews the chemical nature, neuronal actions, receptor subtypes, and therapeutic roles of several transmitters, neurotrophins, and hormones. It focuses on neurotransmitters with important roles in acute and chronic alcohol effects on the brain, such as those that contribute to intoxication, tolerance, dependence, and neurotoxicity, as well as maintained alcohol drinking and addiction.

The behavioral effects of alcohol are produced through its actions on the central nervous system (CNS) and, in particular, the brain. Synaptic transmission—the process by which neurons in the CNS communicate with one another—is a particular target for alcohol actions that alter behavior. Intoxication is thought to result from changes in neuronal communication taking place while alcohol is present in the brain. Tolerance to alcohol involves cellular and molecular adaptations that begin during alcohol exposure; the adaptations develop and diversify with repeated episodes of exposure and withdrawal and are linked to the environment present during exposure. Alcohol dependence develops after several exposure/withdrawal cycles and involves neuroadaptive changes brought about by both the exposure and withdrawal processes. Neurotoxicity produced by alcohol ingestion involves a number of cellular and molecular processes, and neurotransmitters can participate in—and modulate—many of these mechanisms. The actions of alcohol on synaptic transmission also contribute to alcohol-seeking behavior, excessive drinking, and alcoholism. Thus, understanding all of these behavioral actions of alcohol requires some knowledge of neuronal signaling in the brain and, especially, the process of synaptic transmission. This article will focus on the basic processes underlying neuronal communication and review the neuronal actions of several neurotransmitters, neurotrophic factors, and hormones thought to be involved in the neural actions of alcohol. This information, although admittedly incomplete, will provide a foundation for the detailed information on alcohol actions provided in subsequent articles in this issue and in Part 2.

## Neuron-to-Neuron Communication

**Figure 1:**
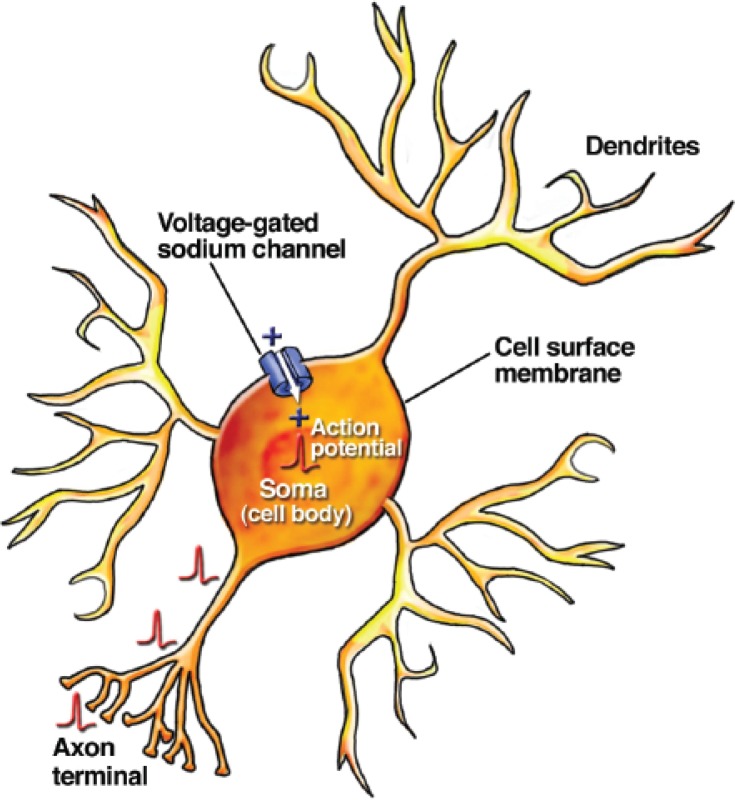
Schematic drawing of a neuron showing dendrites, where neurons receive chemical input from other neurons; soma (cell body); and axon terminal, where neurons communicate information to other cells. Voltage-gated sodium channels in the membrane of the soma, axon, and axon terminal allow positively charged sodium ions to enter the neuron and produce rapid (in milliseconds) conduction of the excitatory action potential to the terminal. This signal stimulates neurotransmitter release at the axon terminal.

Neurons are the cells within the brain that are responsible for rapid communication of information. Although similar to other cells in the body, neurons are specialized in ways that set them apart from other cells and endow them with the properties that allow them to carry out their unique role in the nervous system. The neuron’s shape is one such unique feature. In addition to the cell body, or soma, which is much like that of other cells, neurons have specialized thin branches know as dendrites and axons. Neurons receive chemical input from other neurons through dendrites and communicate information to other cells through axons. Neurons also are “excitable” cells. The neuronal surface membrane contains an abundance of proteins known as ion channels that allow small charged atoms to pass through from one side of the membrane to the other. Some of these channels are opened when the voltage across the cell membrane changes. One subtype of these “voltage-gated” channels allows the neuron to produce a rapid signal known as the “action potential,” which is the fastest form of intracellular electrical signal conduction in biology (see figure 1).

**Figure 2:**
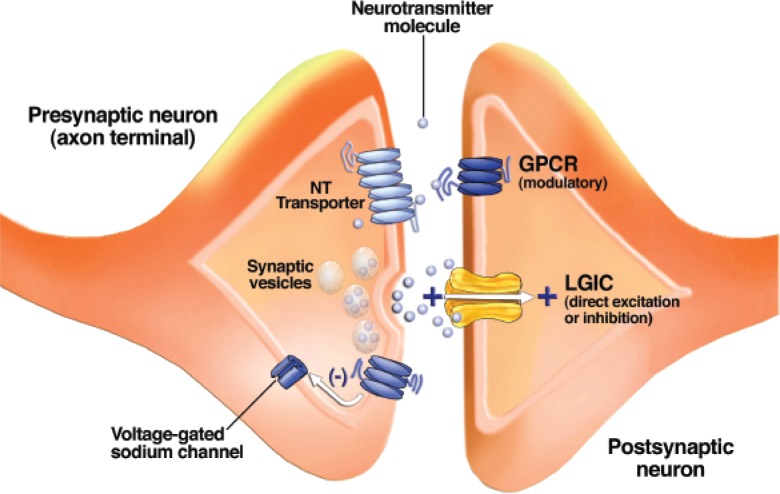
Schematic drawing of a synapse between two neurons. Synaptic vesicles contain a neurotransmitter (NT) and release it when their membranes fuse with the outer cell membrane. Neurotransmitter molecules cross the synaptic cleft and bind to receptors known as ligand-gated ion channels (LGICs) and G-protein–coupled receptors (GPCRs) on the postsynaptic neuron. GPCRs on the presynaptic neuron’s axon terminal alter the function of voltage-gated ion channels and modulate neurotransmitter release. Neurotransmitter transporters remove neurotransmitter molecules from the synaptic cleft so that they can be repackaged into vesicles.

Individual neurons usually are completely separated from one another by their outer cell membranes and thus cannot directly share electrical or chemical signals. The exception to this situation is the so-called electrical synapse, in which ion-conducting pores made from proteins called connexins connect the intracellular compartments of adjacent neurons, allowing direct ion flow from cell to cell (Kandel et al. 2000). This form of interneuronal communication is much less common in the mammalian CNS than chemical transmission and will not be discussed any further. Rather, the focus will be on chemical interneuronal communication involving the release of a neurotransmitter from one neuron, which alters the activity of the receiving neuron. This chemical communication usually occurs at a specialized structure called a synapse, where parts of the two cells are brought within 20 to 50 nanometers of one another (see figure 2). The neuron that releases the chemical is called the presynaptic neuron. A specialized structure at the tip of the axon of the presynaptic neuron, termed the axon terminal, contains small packets known as vesicles, which are filled with neurotransmitter molecules. When an action potential reaches the axon terminal and stimulates a rise in the concentration of calcium, this ion stimulates the vesicle to fuse with the cell membrane and release the neurotransmitter into the small synaptic gap between cells.

The neuron that is acted upon by the chemical is termed the postsynaptic neuron. The neurotransmitter molecules released from the presynaptic vesicles traverse the synaptic gap and bind to proteins, termed neurotransmitter receptors, on the surface membrane of the postsynaptic neuron.

### Neurotransmitter Receptors

Neurotransmitter receptors are divided into two major classes: ligand-gated ion channel (LGIC) receptors and G-protein–coupled receptors (GPCRs). LGIC receptors are proteins specialized for rapid transduction of the neurotransmitter chemical signal directly into an electrical response (Brunton et al. 2005; Kandel et al. 2000) (see figure 3A). One part of the protein is specialized to bind the neurotransmitter molecule. This “binding site” is on the extracellular side of the protein. The part of the protein that is buried within the cell surface membrane forms an ion pore, which is basically a fluid-filled hole in the membrane through which the charged ions can pass (ions cannot pass through lipids or other solid membrane constituents). The time between neurotransmitter binding and opening of the ion pore is on the order of microseconds to milliseconds. Thus, at synapses using ligand-gated channels, the time between action potential depolarization^1^ of the axon terminal and the beginning of the current flowing through the postsynaptic LGIC is a matter of 1 to 2 milliseconds. This type of synaptic transmission produces a rapid and strong influence on postsynaptic neuron function.

Synaptic responses mediated by LGICs are designated as excitatory or inhibitory, depending on whether their net effect is to make it more or less likely that the postsynaptic neuron will fire an action potential (Kandel et al. 2000). In general, strong excitatory synaptic transmission is mediated by LGICs containing an ion pore that allows positively charged ions (i.e., cations) to flow across the membrane. Activation of such a receptor will mainly result in an influx of sodium into the cell, causing the membrane potential to depolarize, bringing it nearer to the action potential threshold. Fast inhibitory synaptic transmission usually is mediated by receptors with channels permeable to negatively charged ions (i.e., anions, usually chloride). When activated, these receptors hyperpolarize the membrane potential and/or fix the membrane potential at voltages below the action potential threshold. The types of neurotransmitters and receptors that subserve these fast excitatory and inhibitory synaptic roles are reviewed below.

GPCRs (shown in figure 3A) are proteins that are specialized for binding the neurotransmitter molecule and subsequently producing intracellular biochemical reactions that can influence a variety of cellular functions (Brunton et al. 2005; Kandel et al. 2000). The GPCR proteins bind directly to small intracellular proteins, known as G-proteins, as their name implies. The G-proteins are so named because they can bind the nucleotides GTP and GDP. When a neurotransmitter binds to the receptor, the exchange of GTP for GDP at the intracellular side of the protein is accelerated, and this causes the G-protein to separate into two parts (the α- and β/γ-subunits), which dissociate from the receptor protein. The liberated α- and β/γ-subunits then are free to interact with different proteins inside the cell. Both types of subunits can act on “effector” proteins to alter cellular biochemistry, physiology, and gene expression. For example, the αS-subunit binds to and activates an enzyme protein called adenylyl cyclase. The function of adenylyl cyclase is to convert the molecule adenosine triphosphate (ATP) to cyclic adenosine monophosphate (cAMP), and the resultant cAMP can act on enzymes that alter the function of cellular proteins through a mechanism called phosphorylation. In another example, free β/γ-subunits can directly bind to and activate a type of ion channel that selectively allows potassium flux across the neuronal membrane (the G-protein–activated inwardly rectifying potassium [GIRK] channel). Activation of this Gβ/γ-GIRK pathway will produce neuronal inhibition, although on a slower timescale than that produced by inhibitory LGICs. In general, the responses produced by GPCRs are slower in onset and longer lasting than those produced by LGICs because several molecular steps are required to get from the receptor protein to the final effector (in contrast to the direct intraprotein signaling within LGICs). The effects of GPCRs on neuronal physiology also often are more subtle than those produced by LGICs because they usually do not directly activate ion current in neurons. Thus, synaptic transmission mediated by GPCRs often is termed neuromodulatory.

### Neurotransmitter Removal

Neurotransmitters are rapidly removed from the synapse after their release. This minimizes the time that they interact with receptors so that short, discrete synaptic signals are produced. At most synapses in the brain, specific proteins known as neurotransmitter transporters mediate this removal (Brunton et al. 2005; Kandel et al. 2000). The transporter proteins reside in the cell surface membrane and actively move the neurotransmitter molecule from the outside to the inside of the cell (see figure 2). In many cases, this uptake occurs at the presynaptic terminal itself, where the neurotransmitter is directly reloaded into vesicles. However, in some cases nonneuronal support cells (i.e., glial cells) also participate in neurotransmitter uptake. Removal of synaptic neurotransmitters also can occur via enzymes that degrade the neurotransmitter to constituent molecules that do not themselves activate receptors.

### Neurotrophins

In addition to neurotransmitters that alter neuronal physiology, intracellular signaling, and gene expression on a relatively fast time scale, certain small chains of amino acids (i.e., peptides) can be secreted by neurons that act as so-called growth factors or neurotrophins. The most widely known neurotrophins are nerve growth factor (NGF) and brain-derived neurotrophic factor (BDNF) and related members of this “cysteine knot” dimeric neurotrophin family that also includes neurotrophin (NT)-3 and NT-4 (Barde 1994; Barde et al. 1982; Levi-Montalcini and Cohen 1960; Reichardt 2006). Other neurotrophins, such as glial-derived neurotrophic factor (GDNF), which acts through the transforming growth factor (TGF)β‚ signaling pathway, are peptides of a different structural class (Unsicker 1996). The neurotrophins are peptides, so their primary amino acid sequence is genetically coded and is subject to alterations in the synthesis of genetic information (i.e., transcription) that can produce different variants of the mature peptide. These peptides also are generated from larger propeptides, and, thus, variations in sequence can occur at the level of posttranslational peptide processing. All of these factors combine to produce a rich variety of neurotrophins in the brain.

Neurotrophins are thought to be secreted from different neuronal structures, including both axon terminals and dendrites (see Altar and DiStefano 1998 for review). Thus, they participate in both “anterograde” signaling from the axon terminal of the presynaptic neuron to the postsynaptic elements of a downstream neuron, as well as “retrograde” signaling, in which release from dendritic elements of the postsynaptic neuron activates receptors on the presynaptic axon terminals.

Receptors for neurotrophins couple to a wide variety of intracellular signaling cascades. The main receptors for the cysteine knot family of neurotrophins are the Trk receptors (Kaplan et al. 1991; reviewed by Chao and Hempstead 1995) (see figure 3B). Each individual neurotrophin binds with highest affinity to a particular Trk receptor (e.g., NGF with TrkA, BDNF with TrkB), but there also are lower affinity interactions that are not as specific (Barbacid 1995; Ip et al. 1993*a*,*b*). Upon neurotrophin binding, the Trk receptors are activated, setting into motion a variety of signaling mechanisms, including the activation of small G-proteins; activation of multifunctional protein kinases, including extracellular signal–regulated kinase (ERK) and the Fyn and Src kinases; activation of lipid-based signaling pathways; and activation of transcription factors that regulate gene expression (Davis 2008). Some of these signaling pathways have effects locally within a particular subcellular compartment. Other signals (e.g., those involving transcription factors) are transmitted to the nucleus. There is evidence that neurotrophin-bound Trk receptors are internalized and translocated to the nucleus, where they can participate in signaling that regulates gene expression (reviewed by Ginty and Segal 2002). Internalization of neurotrophin-bound receptors also is believed to be a major mechanism by which the neurotrophins are removed from the extracellular space and ultimately degraded by intracellular peptidases. The diversity of signaling pathways activated by Trk receptors allows them to participate in a variety of neuronal functions, including not only cell survival and growth but also synaptic plasticity.

Neurotrophins are well known for their ability to support the survival and growth of neurons. For example, the pioneering work of Levi-Montalcini and Cohen (1960) showed that the viability of sympathetic peripheral neurons (those outside the CNS) in culture requires NGF and that this neurotrophin stimulates outgrowth of axons and dendrites (Levi-Montalcini 1987). Neurotrophins are widely expressed within the CNS. For example, BDNF is expressed in a number of brain regions, including many that have been implicated in neural mechanisms of addiction (reviewed in Davis 2008).

### Steroid Hormones

**Figure 4:**
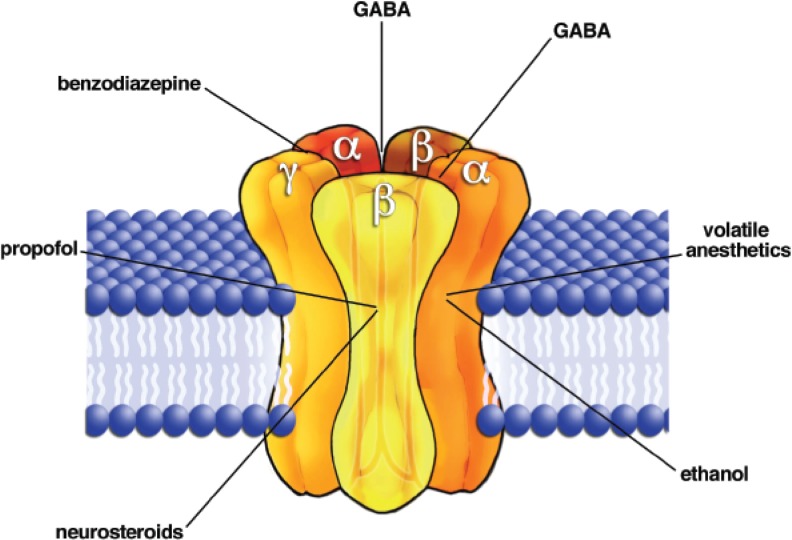
Schematic drawing of the γ-aminobutyric acid receptor (GABA_A_) ligand-gated ion channel complex. The receptor molecule is formed by the confluence of five subunit proteins. In this case, two of the subunits are of the α type, two β and one γ, although many combinations of the 20 known subunits are possible. Globular regions of the protein stick out from the membrane on the extracellular side, and the interfaces between these regions are targets for GABA and for the benzodiazepines and related drugs. The protein domains that span the outer cell membrane are depicted as cylinders. These regions are thought to be targets for general anesthetics (e.g., propofol) neurosteroids, and alcohol. A hole in the middle of the five subunits is the ion conduction pathway, or channel pore.

Steroid hormones––small, complex molecules involved in intercellular communication—are highly lipid soluble and have a variety of actions in the body and brain. For example, corticosteroids are released from the cortex of the adrenal glands located on top of the kidneys in response to external stress and are carried by the bloodstream to their sites of action throughout the body and brain (Brunton et al. 2005). It now is widely appreciated that steroids have two mechanisms of action. The traditional steroid signaling pathway involves an intracellular steroid receptor that resides in the cytosol when unbound and translocates to the cell nucleus when it is bound with the steroid (Brunton et al. 2005; Hayashi et al. 2004) (see figure 3C). The receptor protein then can bind to DNA and directly influence the transcription of a variety of genes. The second type of steroid hormone signaling involves actions on cell surface receptors. For example, derivatives of the sex steroid progesterone interact with the A-type receptors for the neurotransmitter γ-aminobutyric acid (GABA) (i.e., GABA_A_ receptors) to enhance receptor function allosterically by producing a conformation, or shape, change in the receptor (Mitchell et al. 2008) (see figure 4). The so-called neurosteroids that act within the brain can be generated locally within CNS tissue and thus can have paracrine^2^ as well as endocrine actions.

## Receptor Pharmacology

Before discussing specific neurotransmitter molecules and their cognate receptors, it is important to review terminology related to receptor action (see also Brunton et al. 2005). The term agonist refers to a molecule that binds to and activates the receptor. Of course, the neurotransmitter itself is the natural receptor agonist. However, chemists have been able to purify or synthesize other small molecules that mimic the effect of the neurotransmitter. Other small molecules, called competitive antagonists, can bind to the site normally occupied by the neurotransmitter and prevent receptor activation. These competitive antagonist compounds do not activate the receptor but prevent the neurotransmitter from binding. Antagonists also may change the conformation of the receptor such that activation is more difficult. The term *competitive* stems from the fact that the neurotransmitter (or agonist) and the antagonist “compete” for binding to the same part of the receptor, and increasing the concentration of one molecule can overcome the effects of the other. Other types of antagonists bind to parts of the receptor protein that are distinct from the agonist binding site. For example, noncompetitive antagonists react with the receptor and prevent activation in an allosteric manner even when the neurotransmitter molecule binds to the protein. In this case, increasing the concentration of neurotransmitter or agonist cannot overcome antagonist actions, and there is no “competition” for the binding site. Other naturally occurring and synthetic molecules enhance receptor function by binding to a region of the receptor distinct from the neurotransmitter/agonist binding site and improving the efficiency of receptor activation. These compounds generally are known as allosteric enhancers of receptor function.

Receptor agonists, antagonists, and allosteric modulators are used as pharmaceutical treatments for a variety of neurological and psychiatric disorders (Brunton et al. 2005). For example, a small molecule called baclofen can control certain types of movement spasticity through its agonist action at the B-type receptor for the neurotransmitter GABA (Bowery 2006). Many of the major antipsychotic drugs used in schizophrenia treatment, such as Haldol^®^, are competitive antagonists at the type 2 receptor for the neurotransmitter dopamine (Kapur et al. 2006). In addition, Valium^®^ (also known as diazepam), Ambien^®^ (also known as zolpidem), and related antianxiety and sleep aid drugs are allosteric enhancers of the GABA_A_ receptor (Sanger 2004), which is the other major receptor for this neurotransmitter. Indeed, neurotransmitter receptors are the predominant targets for therapies aimed at treatment of brain disorders.

## The Neurotransmitter Molecules

Many small organic molecules serve as neurotransmitters in the brain. For example, amino acids such as glutamate and glycine, which are well known as constituents of proteins, also act as neurotransmitters (Kandel et al. 2000). Histamine, a molecule that has a prominent role in inflammation and infection in the body, also is a neurotransmitter (Haas and Panula 2003). A variety of peptides also have been found to act as neurotransmitters (Kandel et al. 2000). A review of all neurotransmitters is beyond the scope of this article. Rather, the sections that follow focus on those neurotransmitters whose actions are most strongly implicated in alcohol intoxication, tolerance, dependence, and addiction. This discussion is organized according to the neurotransmitters’ proposed roles within the chronology of alcohol actions. Those neurotransmitters thought to be most heavily involved in intoxication are discussed first, followed by those involved in chronic alcohol effects. The final section addresses those neurotransmitters that do not appear to be direct targets for the neural actions of alcohol but which may be involved in alcohol abuse and addiction and are potential pharmacotherapeutic targets.

## GABA

GABA mediates the majority of fast synaptic inhibition in the brain, specifically through activation of GABA_A_ receptors. Like glutamate, GABA also is found in all brain regions. The intrinsic ion channel contained in the GABA_A_ receptor protein is permeable to Cl^−^ and other anions (Kandel et al. 2000). Activation of the receptor can hyperpolarize neurons through the influx of negative charges at membrane potentials below the threshold for action potential generation. This inhibition generally counteracts the effect of glutamate and other depolarizing, excitatory synaptic influences. The amino acid glycine produces a similar action in the spinal cord and posterior parts of the brain.

Several subtypes of GABA_A_ receptors exist; they are formed by the confluence of individual “subunit” proteins (see figure 4). Each subunit has a slightly different amino acid sequence, and there are 20 subunits in all in the mammalian brain (Sieghart and Sperk 2002). Thus, a variety of different types of GABA_A_ receptors are made in different brain neurons. GABA_A_ receptors are present both at the synapse and on postsynaptic membranes distant from synapses. These latter “extrasynaptic” GABA_A_ receptors are sensitive to very low levels of extracellular GABA, and thus they often produce a continuous inhibitory tone that helps to set the resting potential of certain neurons (Stell et al. 2003).

The GABA_A_ receptor channel contains numerous sites for allosteric modification. Perhaps the best known among these is the “benzodiazepine” binding site (Sanger 2004; Sigel 2002). This site resides on the extracellularly exposed part of the protein at a site distinct from the agonist binding region. Many compounds, such as Valium, that bind to this site have potent allosteric enhancing effects on the receptor. Other compounds can have an antagonist action at this site and will prevent allosteric enhancement by benzodiazepines without altering the response of the receptor to GABA (Mohler 1983). The diversity of GABA_A_ receptor subtypes has given rise to different benzodiazepine effects on different receptors and different brain neurons, providing multiple possibilities for pharmaceutical development.

Drugs that target the GABA_A_ receptor have been in widespread use for treatment of disorders ranging from anxiety to epilepsy (Mohler 2006; Sigel 2002). The majority of general anesthetics currently in use produce their actions predominantly through enhancing GABA_A_ receptor function (Hemmings et al. 2005). Almost all of these therapeutic drugs either directly activate the GABA_A_ receptor or activate the benzodiazepine site to produce allosteric enhancement of the receptor, as mentioned above.

GABA also can act through a GPCR, the aforementioned GABA_B_ receptor. This receptor acts to inhibit neuronal activity in two ways (Bowery et al. 1990). First, activation of GABA_B_ receptors leads to a direct G-protein–mediated activation of the GIRK-type potassium channel. This channel can hyperpolarize neurons and counteract synaptic excitation. This type of GABA_B_-mediated modulation is found in many postsynaptic neuronal elements throughout the brain. GABA_B_ receptors also are found on presynaptic terminals of neurons in many brain regions (Bowery et al. 1990). When activated, these receptors inhibit neurotransmitter release via mechanisms that involve inhibition of voltage-gated calcium channels whose function is necessary for proper release. Presynaptic GABA_B_ receptors are found on the axon terminals of both GABAergic and glutamatergic neurons, and thus the net effect of receptor activation can be either disinhibitory or inhibitory depending on which receptors are activated.

GABAergic transmission is a sensitive target for both the acute and chronic effects of alcohol (reviewed in Lovinger and Homanics 2007; Siggins et al. 2005). Acute alcohol can produce allosteric enhancement of the function of GABA_A_ receptors, although this effect is not observed at all GABA_A_ receptors, and there is some debate as to which receptor subtypes are most sensitive to alcohol (reviewed in Lovinger and Homanics 2007). Researchers have observed effects at concentrations as low as the low millimolar range (the levels reached with ingestion of a single drink), suggesting that even some aspects of low-dose intoxication might involve enhanced GABA_A_ receptor function. Acute ethanol also enhances the release of GABA at a number of synapses in the brain (Siggins et al. 2005). The mechanisms of this presynaptic enhancement are just beginning to be explored. One interesting set of studies indicates that activation of presynaptic GABA_B_ receptors prevents alcohol potentiation of GABA release and can bring about a “tolerance” to the alcohol action (Ariwodola and Weiner 2004). Potentiation of GABAergic synaptic transmission appears to contribute to a number of aspects of acute alcohol intoxication, including motor incoordination, anxiety-reducing effects, sedation, and the internal cues that signal intoxication (Lovinger and Homanics 2007; Siggins et al. 2005; Vengeliene et al. 2008).

The brain’s GABAergic system also shows marked changes following chronic alcohol exposure. Some of these changes are likely to be adaptations to the acute alcohol actions that potentiate GABAergic transmission. The best-characterized changes involve alterations in the subunits that make up the GABA_A_ receptor (Kumar et al. 2004), which alter the efficacy and timing of inhibitory synaptic transmission. Research also shows that chronic alcohol exposure can produce decreases and increases in GABA release in different brain regions (reviewed in Weiner and Valenzuela 2006). The predominant effect of these chronic alcohol effects is to make the brain hyperexcitable during withdrawal from chronic alcohol exposure. This can produce effects such as heightened anxiety and even overt seizures during withdrawal (Krystal et al. 2006; Kumar et al. 2004). Benzodiazepines commonly are used to treat alcohol withdrawal because of their effectiveness in controlling these aspects of hyperexcitability (Krystal et al. 2006). Drugs that target GABAergic transmission also have been touted as potential pharmacotherapeutic treatments for alcohol abuse and alcoholism (Koob 2004; Krystal et al. 2006), but, as yet, no drugs that explicitly and specifically target GABAergic mechanisms are in clinical use for this purpose.

## Glutamate

Glutamate is the major excitatory neurotransmitter in the mammalian brain. Fast transmission mediated by this neurotransmitter accounts for synaptic excitation of most, if not all, brain neurons (Kandel et al. 2000), and thus glutamate is found throughout the brain. It is not surprising, therefore, that glutamate has key roles in a wide variety of brain functions. The fast synaptic excitation produced by glutamate involves the activation of three major subtypes of LGICs, termed the AMPA (α-amino-3-hydroxy-5-methyl-4-isoxazolepropionic acid)^3^-, kainate-, and NMDA (*N*-methyl-d-aspartic acid)^4^-type receptors based on the synthetic agonists that best activate each receptor (Kandel et al. 2000). At most excitatory synapses, glutamate released from presynaptic vesicles crosses the synapse and binds to AMPA-type glutamate receptors. The binding of glutamate to AMPA receptors directly activates the ion pore intrinsic to this protein and produces a rapid depolarization of the postsynaptic neuron that increases the likelihood that this neuron will fire an action potential. Thus, timely and accurate communication within brain circuits depends to a large extent on proper AMPA receptor activation.

The function of NMDA-type glutamate receptors is more complicated. When glutamate binds to the NMDA receptor, activation of the intrinsic ion pore is favored, as is the case for AMPA receptors and other LGICs. However, at membrane potentials near the resting potential (e.g., −60 to −70 mV), the ion pore of the NMDA receptor is occluded by magnesium ions (Kandel et al. 2000). Magnesium is present at millimolar concentrations in the extracellular solution and binds to a site within the NMDA receptor ion pore that becomes accessible very shortly after the ion pore opens. This magnesium pore blocking action is stronger at more negative membrane potentials and is thus reduced as the membrane potential becomes more depolarized. In practice, at a glutamatergic synapse that contains both AMPA and NMDA receptors, the initial action of glutamate will be to activate AMPA receptors. This will depolarize the cell because of the influx of cations through the AMPA receptor ion pore. If the depolarization is sufficiently strong and glutamate still is present, the NMDA receptor pore will become unblocked, and ion flux through this receptor-channel also can participate in activating the postsynaptic neuron.

The AMPA and NMDA receptors have central roles in producing long-lasting changes in synaptic function that are thought to represent molecular mechanisms of learning and memory (Kandel et al. 2000). One such change, named long-term potentiation, is a long-lasting increase in the strength of signaling at glutamatergic synapses that mediate fast excitatory synaptic transmission. When these synapses are activated repeatedly and perseveratively (e.g., by high-frequency synaptic input) the repeated activation of AMPA receptors will depolarize the postsynaptic neuron sufficiently to allow for ion current to flow through NMDA receptors. The ion pore of the NMDA receptor is especially permeable to calcium ions that can activate a variety of intracellular signaling cascades, including protein kinases. Strong NMDA receptor activation sets into motion a sequence of molecular events that result in the insertion of additional AMPA receptors at the synapses, thus strengthening synaptic transmission (Kandel et al. 2000). There now is abundant evidence that this NMDA receptor–dependent long-term potentiation is involved in a variety of types of learning in the brain (Robbins and Murphy 2006).

Glutamate also can activate GPCRs known as metabotropic glutamate receptors (mGluRs) (Kandel et al. 2000). Eight mGluRs are known to exist and can be separated into three groups termed I, II, and III (Coutinho and Knopfel 2002). The group I mGluRs couple through the G_q_-type G-protein subtype to activate enzymes called phospholipases. These receptors mainly are on postsynaptic structures, where they serve to activate intracellular signaling that modifies ion channel function and biochemical processes including the generation of second messengers^5^ from cell membrane lipids. The group II and III mGluRs couple to G_i/o_-type G-proteins. Members of these latter two mGluR subgroups reside predominantly on presynaptic axon terminals, where they act to inhibit neurotransmitter release (Coutinho and Knopfel 2002).

Therapeutic uses for glutamate receptor–targeted drugs are a matter of intensive research at present. Ketamine is an antagonist of NMDA receptor function that has been used as a general anesthetic in animals and, in some cases, humans (Sinner and Graf 2008). Recent studies suggest that group II mGluR agonists may be useful in the treatment of psychosis (Patil et al. 2007).

Acute alcohol exposure generally inhibits glutamatergic synaptic transmission (Siggins et al. 2005; Woodward 2000). At most synapses in the CNS, NMDA receptor function is the aspect of glutamatergic synaptic transmission that is most sensitive to alcohol inhibition (Woodward 2000). Alcohol also inhibits synaptic plasticity that requires NMDA receptor activation, and this effect may contribute to the memory-impairing effects of alcohol. Alcohol interactions with NMDA receptors also may contribute to its intoxicating effects (Krystal et al. 2003).

Chronic alcohol exposure results in an increase in NMDA receptor number and function that likely is a compensatory change induced by the inhibitory effect of acute alcohol (Fadda and Rossetti 1998). This increased NMDA receptor function is thought to contribute to withdrawal hyperexcitability and perhaps also to alcohol-induced neuronal damage. A hyperglutamatergic state also may result from chronic alcohol exposure, and there is growing evidence that decreasing this excessive glutamatergic transmission might be helpful in reducing relapse to alcohol drinking (Vengeliene et al. 2008). Indeed, a few drugs that target glutamatergic transmission, either directly or indirectly, have been used for treatment of alcoholics or currently are being tested for their efficacy in reducing drinking and/or relapse in alcoholics (Krystal et al. 2003).

## Dopamine

**Figure 5:**
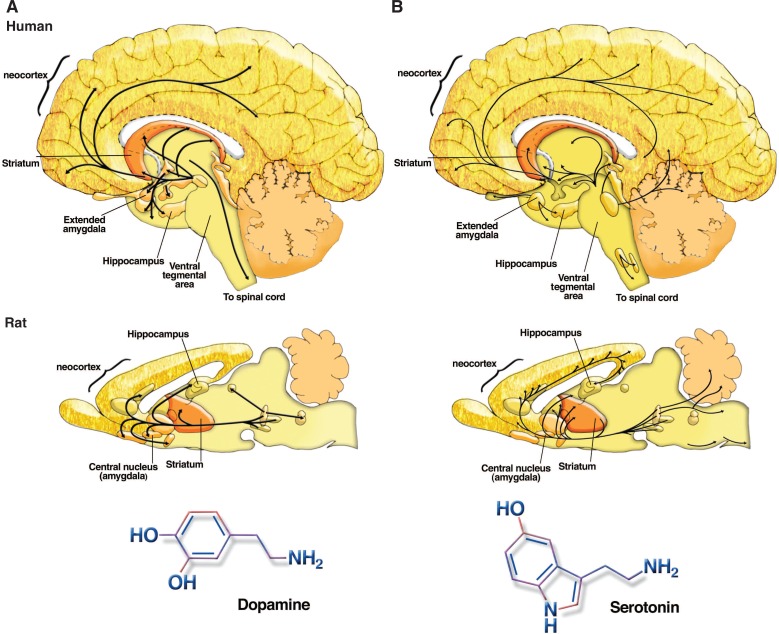
Neurotransmitters with discrete localization within the brain. **A)** The chemical structure of the monoamine neurotransmitter dopamine and a schematic drawing of the localization of dopamine-containing neurons in the human and rat brain and the sites where dopamine-containing axons are found. **B)** The chemical structure of the monoamine neurotransmitter serotonin and similar brain map showing locations of serotonin-containing cells and their axons.

The neurotransmitter dopamine has been widely implicated in a variety of neuronal functions, including brain mechanisms of reward, evaluation of environmental stimuli, general behavioral activity levels, and disorders such as Parkinson’s disease and schizophrenia (Brunton et al. 2005; Kandel et al. 2000). Considering that dopamine is made by very few cells in the brain and acts mainly within a subset of brain regions (see figure 5A), this neurotransmitter seems to have a disproportionately large impact on brain function.

Dopamine acts exclusively via activation of GPCRs and thus is a pure neuromodulator. There are five identified dopamine receptors that fall into two subclasses: those that activate G_s_/G_olf_-type G-proteins (D1 and D5) and those that activate G_i/o_-type G-proteins (D2-4) (Lachowicz and Sibley 1997). In general, the two classes of receptors produce separate, and often opposing, effects on neuronal physiology. For example, D1-like receptors enhance sodium channels in neurons from the brain region known as the striatum, whereas D2 receptors inhibit these same channels (Surmeier and Kitai 1993). However, there are situations in which the two dopamine receptor subtypes appear to work together or even produce synergistic actions (Lee et al. 2004). Within the striatum, D1 and D2 receptors mainly are segregated onto two different types of neurons (Gerfen 1992). The medium spiny neurons provide the only source of output from this brain region. Half of these neurons connect to a brain region called the substantia nigra pars reticulata, forming the so-called “direct” pathway that tends to activate the cortex. The other half connect to a brain area called the globus pallidus internal segment and thus form the “indirect” pathway that tends to dampen cortical output. The coordination of these two pathways determines one’s ability to initiate actions and control action sequences. Thus, dopamine, working through these two receptor subtypes, has key roles in controlling performance of actions, including those affected by intoxication and those involved in addiction.

Dopaminergic transmission is targeted by many drugs, including both legal prescription medications and illegal drugs of abuse. Perhaps the most widely known brain disorder involving the brain dopaminergic system is Parkinson’s disease. This debilitating and ultimately lethal neurological disorder arises from the death of dopaminergic neurons and the resultant loss of brain dopamine (Kandel et al. 2000). The most common therapy for this disease involves dopamine replacement by treatment with the dopamine precursor l-Dopa (Stacy and Galbreath 2008), which is taken up by the brain and used to make more dopamine. Dopamine receptor agonists also are sometimes used as an adjunct therapy with l-Dopa (Stacy and Galbreath 2008). Agonists of the D_2_ class of dopamine receptors are used in the treatment of the movement disorder known as restless legs syndrome (Winkelman et al. 2007). Dopamine receptor- and transporter-targeted drugs also have a large role in the treatment of other neurological and neuropsychiatric disorders. The majority of antipsychotic drugs have potent actions at the D_2_-like dopamine receptors, although they certainly have effects on other targets that could contribute to their therapeutic efficacy (Kapur et al. 2006). Drugs that block the dopamine transporter, such as methylphenidate (Ritalin^®^) are used in the treatment of attention deficit hyperactivity disorder (ADHD) (Arnsten 2006) (note the potential abuse liability of this class of drugs, as discussed below) (Faraone and Wilens 2007). Antagonists for D_2_ dopamine receptors also are used clinically to reduce vomiting in circumstances such as chemotherapy (Reddymasu et al. 2007).

Cocaine, amphetamine, and other “stimulant” drugs block or reverse the action of the dopamine transporter (Amara and Sonders 1998). The net effect of these drugs is to increase levels of dopamine within the synapse. Although these drugs act on other molecular targets, the evidence is fairly convincing that it is the effect on dopaminergic transmission that accounts for the majority of the intoxicating and addictive actions of these drugs. Most other drugs of abuse also influence the brain dopaminergic system. Nicotine stimulates the activity of the dopaminergic neurons themselves (Pidoplichko et al. 1997) and also can activate dopamine release from axon terminals (Grady et al. 2007). Morphine and other opiate drugs depress the activity of GABAergic interneurons and, through this effect, indirectly increase the activity of dopaminergic neurons (Johnson and North 1992). Alcohol also increases the activity of these neurons, likely via both direct actions on the neurons and indirect actions through other neurons (Brodie et al. 1999; Ericson et al. 2008; Gessa et al. 1985; Okamoto et al. 2006).

Dopaminergic transmission has been implicated in the actions of almost all drugs of abuse, and transmission mediated by this neurotransmitter is altered by alcohol exposure in both the acute and chronic phases (reviewed in Vengeliene et al. 2008). The acute alcohol-induced increase in firing of dopaminergic neurons appears to drive increases in extracellular dopamine levels in the brain regions to which these neurons project (reviewed in Gonzales et al. 2004). Imaging of dopamine receptors in the human brain also suggests that acute alcohol exposure alters dopamine levels in key brain structures (reviewed in Wong et al. 2003). These effects may contribute to the process by which animals encode the reinforcing value of alcohol.

As animals learn to consume alcohol in the laboratory, increases in brain dopamine levels become associated with stimuli that predict access to alcohol (Gonzales et al. 2004). Thus, dopamine also plays a role in learning about environmental contexts that encourage drinking. Chronic alcohol consumption can lead to a hypodopaminergic state that motivates the drinker to seek alcohol in order to restore the desired levels of the neurotransmitter (Volkow et al. 2007). However, despite these findings, pharmacotherapies aimed at the dopaminergic system have not shown particular efficacy in reducing alcohol drinking either in animal models or in humans with alcohol abuse disorders. Perhaps the development of drugs with greater specificity for certain subtypes of dopamine receptors will prove more efficacious in this context.

## Adenosine

Adenosine is a purine nucleoside (a compound with a nitrogen-containing base linked to a sugar molecule) that is produced during nucleic acid metabolism. This compound also participates in a number of types of cell–cell communication, including synaptic transmission in all regions of the nervous system. Adenosine primarily is a neuromodulatory transmitter and produces its actions via the activation of two main types of GPCRS, the A1 and A2a adenosine receptors (Fredholm et al. 2005). Other adenosine receptors exist but are present only in small quantities within the CNS.

Activation of A1 adenosine receptors in turn activates G_i/o_ class G-proteins. In general, these G-proteins activate GIRK potassium channels, inhibit voltage-gated calcium channels, inhibit adenylyl cyclase, or activate phosphorylation of certain intracellular protein kinase enzymes (Fredholm et al. 2005). Adenosine A2a receptors activate G_s_ or G_olf_–type G-proteins (Fredholm et al. 2007). These G-proteins stimulate adenylyl cyclase to enhance production of the second messenger cyclic AMP (Fredholm et al. 2007).

After producing its actions on synaptic adenosine receptors, the neurotransmitter is transported back into cells via a cell membrane neurotransmitter transporter protein known as the adenosine transporter (Fredholm et al. 2005). Adenosine also can be metabolized by enzyme proteins known as adenosine deaminase and adenosine kinase (Fredholm et al. 2005). The transporter and enzymes regulate the duration of adenosine signals within the synaptic cleft. Inhibition of either process can prolong the time that the neurotransmitter is present in the synapse and, consequently, the duration of activation of the adenosine receptors.

The behavioral effects of modifying brain adenosinergic communication are very familiar to many of us. Caffeine acts as an adenosine receptor antagonist (Dunwiddie and Masino 2001). The major behavioral effects of caffeine, including enhanced activity and focused attention, appear to involve inhibition of the function of both A1- and A2a-type adenosine receptors, although locomotor stimulation by caffeine is predominantly because of A2a antagonism.

Acute alcohol exposure increases adenosine signaling in cell lines of neural origin (Nagy et al. 1990). This effect appears to involve inhibition of a nucleoside transporter that normally produces rapid uptake of adenosine into cells. This inhibitory action increases extracellular adenosine levels and prolongs the duration of adenosine signaling to the cell. The role of these changes in adenosinergic transmission in acute intoxication is not clear, although removing the alcohol-sensitive nucleoside transporter in mice alters intoxication and alcohol intake (Choi et al. 2004). Chronic alcohol exposure causes a compensatory downregulation of A2a adenosine receptors and decreases in the adenylyl cyclase signaling by these receptors (Diamond et al. 1991). These receptors predominantly are found in brain regions involved in neural processes important for reward, habit formation, and addiction (Jarvis and Williams 1989). Thus, alterations in adenosine signaling in these brain regions could contribute to alcohol addiction (Choi et al. 2004; Naassila et al. 2002).

## Serotonin

The neurotransmitter serotonin (also known as 5-hydroxytryptamine or 5-HT) is a close molecular relative of dopamine that belongs to the family of neurotransmitters known as monoamines (Kandel et al. 2000). Like dopamine, serotonin is made by small discrete clusters of neurons located at the base of the brain (see figure 5B). These serotonergic neurons connect to other neurons located throughout the CNS, including neurons in the cerebral cortex and other forebrain structures. Thus, serotonin has the capacity to influence a variety of brain functions including sensations related to environmental stimuli, pain perception, learning and memory, and sleep and mood (Kandel et al. 2000). The role of serotonin in mood control has received considerable attention in both the laboratory and the clinic, as selective serotonin reuptake inhibitors (SSRIs) such as Prozac^®^ are the most widely prescribed drugs for depression and other mood disorders (Brunton et al. 2005; Kandel et al. 2000).

The majority of serotonin actions in the brain occur via activation of GPCRs. The human brain has 15 serotonin-activated GPCRs that activate a wide variety of G-protein subtypes (Kitson 2007). Thus, serotonin can produce neuromodulatory effects that tend to either increase or decrease neuronal output. Different subtypes of these GPCRs are found on presynaptic and postsynaptic neuronal structures in different brain regions. By activating these receptors, serotonin can enhance or inhibit neurotransmitter release at certain synapses and can produce slow hyperpolarizing or depolarizing synaptic responses at others. Intracellular signaling via serotonin-activated GPCRs also can influence the function of intracellular enzymes as well as alter gene expression.

Serotonin also can activate a single type of LGIC-type neurotransmitter receptor, the so-called 5-HT_3_ receptor (Thompson and Lummis 2007). This receptor contains a channel that is permeable to positively charged cations and thus produces fast activation of neurons when bound to serotonin. One interesting facet of the action of this receptor is that often it resides on axon terminals that contain and release GABA. Thus, activation of these presynaptic 5-HT_3_ receptors leads to a rapid release of GABA that will then inhibit downstream neurons.

In addition to the use of SSRIs for neuropsychiatric therapy, as mentioned above, a variety of other therapeutic uses exist for serotonergic drugs. The SSRIs produce their actions via inhibition of the serotonin transporter protein, as their name implies. Buspirone is an anxiety-reducing drug that acts on the 5-HT-1_A_ receptor (Barrett and Vanover 1993), and use of this drug has been suggested for other disorders. The 5-HT_3_ antagonists routinely are used to reduce nausea and vomiting resulting from chemotherapy and can be used for similar purposes following surgical anesthesia, and these drugs also have been used for treatment of irritable bowel syndrome (Thompson and Lummis 2007).

The brain serotonergic system also is the target of psychoactive drugs, including many that are illegal. The largest class of hallucinogenic drugs, including LSD, mescaline, and psilocybin, are all partial agonists of the 5-HT_2_ receptor subtypes, and the 5-HT_2A_ receptor is implicated in the effects of these drugs (Fantegrossi et al. 2008). Several amphetamine derivatives, such as 3,4-methylenedioxymethamphetamine (MDMA, also known as ecstasy), alter serotonin transporter molecules and increase synaptic serotonin levels (McKenna and Peroutka 1990). This may account for their sensory-enhancing effects.

Acute alcohol has mixed effects on serotonergic transmission (reviewed in Lovinger 1997). A slowing of serotonergic reuptake is observed (Daws et al. 2006), but this does not appear to be because of impairment of the serotonin transporter targeted by SSRIs. Alcohol also potentiates the function of the 5-HT_3_ receptor (Lovinger 1999).

Chronic alcohol exposure has been demonstrated to interact with various aspects of serotonergic transmission that could alter anxiety and affect (reviewed in Lovinger 1997). Based on the well-known role of serotonin in neural mechanisms underlying mood and stress responses, pharmacotherapies aimed at the serotonergic system have long been touted as potential treatments for alcoholism (reviewed in Ait-Daoud et al. 2006). Treatments with SSRIs are efficacious in reducing alcohol intake in laboratory animals (LeMarquand et al. 1994; Pettinati et al. 1996) but have had mixed success in humans (Ait-Daoud et al. 2006; Naranjo and Kadlec 1991). It is possible that these drugs might work best in patients with comorbid depression. The 5-HT_3_ antagonist ondansetron reduces relapse in alcoholics, particularly in those with early onset (reviewed in Ait-Daoud et al. 2006).

## Opioids and Other Peptides

Peptides have long been known to function as hormones within the body and to participate in synaptic communication in the brain (Brunton et al. 2005; Kandel et al. 2000). All of the known peptide actions in the brain are neuromodulatory, and thus these agents act through GPCRs. Many peptides act as neurotransmitters (i.e., the so-called neuropeptides); this section will focus on a few neuropeptides that have been implicated in alcohol actions on the brain.

Most of the neurotransmitter molecules discussed above are synthesized within the axon terminal itself and are then delivered into the vesicles waiting near the presynaptic release sites. Peptide neurotransmitters, however, normally are synthesized in the neuronal cell body, some distance from the site of release (Kandel et al. 2000). The neuropeptides then are packaged into vesicles and transported inside the vesicle to the axon terminal. Thus, it is somewhat easier to deplete the store of neuropeptide transmitters in a given axon terminal, and it can only be refilled after transport from the cell body.

The opioid peptides are some of the most widely known neuropeptides. These include β-endorphin (which consists of 31 amino acids), dynorphin (13 amino acids), and the enkephalins (5-amino-acid peptides that end in either methionine [met-enkephalin] or leucine [leu-enkephalin]) (Kandel et al. 2000). The notoriety of these peptides stems from the fact that they serve as agonists for the receptors that also are activated by morphine, heroin, and the other opiate drugs. These receptors are known as opiate receptors, and there are three different subtypes designated the μ, δ, and κ receptors (Connor and Christie 1999). In addition, a peptide known as nociceptin or orphanin FQ has actions at a separate opiate-like receptor called the ORL-1 receptor (Connor and Christie 1999). All of these receptors preferentially couple to G_i/o_-type G-proteins and thus generally are known to inhibit neurotransmitter release and reduce the activity of neurons (Williams et al. 2001).

Opioid peptides are found in many regions of the nervous system. Like other neuropeptides, the opioids usually are stored in and released from large vesicles different from those that contain the small molecules (e.g., glutamate or GABA). These opioid peptide–containing vesicles often are found in the same axon terminals with the smaller diameter, small molecule–containing vesicles, and thus many neurons have the capacity to release both small molecule and opioid peptide neurotransmitters (Kandel et al. 2000). Opiate receptors are found on both presynaptic and postsynaptic structures (Williams et al. 2001). The termination of transmission mediated by opioid peptides involves peptidases, which are specific enzymes that catalyze the degradation of the peptide into its constituent amino acids (Schwartz et al. 1981). These amino acids are then taken back into cells via amino acid transporters, where they can be used for future peptide or protein synthesis.

Opiate receptors have been targeted for a number of therapeutic uses. Morphine, heroin, fentanyl, and other powerful opiate receptor agonists have long been used for pain reduction (Brunton et al. 2005). The opiate receptor partial agonist methoadone is a well-known and effective treatment for addiction to opiate drugs (Brunton et al. 2005), basically substituting for morphine or heroin to prevent withdrawal symptoms without having the same debilitating effects.

Of course, opiate agonists are among the drugs with the strongest abuse/addiction liability (Brunton et al. 2005). Injectable heroin is perhaps the best-known addictive opiate and continues to be a problem for people worldwide. However, abuse of powerful synthetic opiates taken in pill form, such as Oxycontin^®^ (also known as Oxycodone), has been on the rise in recent years (Compton and Volkow 2006). Another psychoactive drug, the plant derivative known as salvanorin A, acts as an agonist at κ-opioid receptors (Roth et al. 2002). When ingested, it produces a relatively short-lasting disorientation, such that the user becomes unaware of his/her location in space and time.

Acute alcohol alters endogenous opioid peptides and opiate receptors (Charness 1989; Gianoulakis 1989). However, the contribution of these actions to intoxication remains unclear. Chronic alcohol exposure also alters brain opiatergic systems (Charness 1989; Gianoulakis 1989). Interestingly, opiate receptors have emerged as useful targets for pharmacotherapeutic treatment of alcohol use disorders (reviewed in O’Brien 2005; O’Malley and Froehlich 2003). Use of the general opiate receptor antagonist naltrexone is approved for the treatment of alcoholics. This compound also reduces alcohol drinking in rodents, apparently via blockade of the μ-type opiate receptor (Altshuler et al. 1980; Gonzales and Weiss 1998; Krishnan-Sarin et al. 1998). The mechanism of action may involve a reduction of endogenous opioid peptide actions that normally promote increases in dopamine release (Gonzales and Weiss 1998).

Many other neuropeptides originally found to act as hormones also have been found to act as neurotransmitters. Corticotrophin-releasing hormone (CRH) was originally known for its role in the pituitary gland, where it stimulates a cascade of molecular processes that ultimately leads to the release of the cortisone-like steroid hormones (e.g., corticosteroids) (Brunton et al. 2005). Within the brain, CRH can communicate signals related to stress, mood, and changes in other bodily functions (Reul and Holsboer 2002). The cellular release of this neuropeptide is stimulated by alcohol (Nie et al. 2004) as well as by exposure to stressful stimuli. Mounting evidence suggests that CRH and its receptors participate in the interactions between stress and alcohol, including increased drinking or relapse to drinking following stressful events (Heilig and Koob 2007).

A variety of other neuropeptides have been implicated in brain responses to alcohol and alcohol drinking behavior (see Thorsell 2007 and Wurst et al. 2007 for more information).

## Endocannabinoids and Other Lipid-Derived Neuromodulators

Molecules derived from the chemical modification of the lipids found in neuronal membranes are another class of neuromodulatory substances. Throughout the body, lipid-derived molecules are known to have paracrine actions. The lipid metabolites known as prostaglandins are perhaps the best-known examples of such paracrine agents (Brunton et al. 2005). It is worth noting that lipid-derived compounds do not have to be released from synaptic vesicles. Unlike other neurotransmitters, they usually escape from neurons directly through the cell surface membrane.

Many of these lipid-derived agents are involved in synaptic communication. Endocannabinoids (a contraction of endogenous cannabinoids) are an example (Chevaleyre et al. 2006). Arachidonoyl ethanolamide (AEA) and 2-arachidonoyl glycerol (2-AG) are two compounds that can be produced upon breakdown of membrane lipids that contain the fatty acid known as arachidonic acid. These compounds are produced throughout the brain and body and have been implicated in a number of physiological functions. Endocannabinoids have an intriguing retrograde signaling action at synapses (Alger 2002). In other words, synaptic communication by these compounds generally occurs in a direction opposite to that of traditional neurotransmitters. The endocannabinoids often are produced by postsynaptic neuronal elements and act on their cognate receptors, cannabinoid 1 (CB1) receptors, which are found almost exclusively on presynaptic axon terminals in the brain. This signaling requires that the compound traverse the synapse in a backward, or retrograde, direction. This action of endocannabinoids has now been found throughout the nervous system.

The CB1 receptor is a GPCR that links to G_i/o_-type G-proteins. The most common effect of activating this receptor is inhibition of neurotransmitter release (Lovinger 2008). Thus, retrograde endocannabinoid signaling results in decreased release of several types of neurotransmitters, including both GABA and glutamate. Endocannabinoid effects are therefore disinhibitory (relief from GABAergic inhibition) or inhibitory (decreased glutamatergic excitation) depending on the brain region and synapses in which they act. The presence of endocannabinoids in the synapse often leads to inhibition of neurotransmitter release that persists for as long as the endocannabinoid is present (Chevaleyre et al. 2006; Lovinger 2008). Endocannabinoids also can trigger a long-lasting depression of neurotransmitter release called long-term synaptic depression (LTD) (Chevaleyre et al. 2006; Lovinger 2008). This depression is set into motion by endocannabinoid activation of CB1 receptors but does not require sustained CB1 activation for its long-term maintenance. Accumulated evidence suggests that the synaptic depression produced by retrograde endocannabinoid signaling has key roles in brain mechanisms of learning and memory as well as addiction (Lovinger 2008).

The term “cannabinoid” within the name of these compounds reflects that fact that the endocannabinoids have something in common with drugs derived from the Cannabis *sativa* plant, such as marijuana. Indeed, the CB1 receptor is itself the major target for Δ-9tetrahydrocannabinol (Δ-9THC), the primary psychoactive ingredient in cannabis-derived drugs (Brunton et al. 2005). The endocannabinoids produce much milder and shorter-lasting versions of the effects produced by cannabinoids in the brain, including relief from anxiety, relief from pain, actions that affect movement initiation, balance and coordination, and effects on cognition (Lovinger 2008; Piomelli et al. 2000). Knowledge of the neuronal effects of Δ-9THC provides a great deal of information on the effects of specific CB1 agonists. Indeed, a number of synthetic agonists have been developed for this receptor, and they produce strong intoxication, movement impairment, decreased learning and short-term memory, pain relief, and, at high doses, a loss of movement known as catalepsy (Howlett 1995). At low-to-moderate doses, these compounds also stimulate appetite, a well-known effect of marijuana and other illegal cannabis-derived drugs (Di Marzo et al. 2001).

Antagonists for the CB1 receptor also have neural actions, and there is increasing evidence of the therapeutic usefulness of these compounds. Studies in laboratory animals indicate that CB1 antagonists reduce feeding and the metabolic changes that often accompany obesity (Di Marzo et al. 2001; Ravinet Trillou et al. 2003). The CB1 antagonist known as Acomplia^®^, Rimonabant, or SR141716 already is in use in Europe for treatment of obesity and the associated metabolic disorder. Antagonists for this receptor also reduce self-administration of a number of drugs of abuse, most notably nicotine and alcohol (Maldonado et al. 2006; Wang et al. 2003). Indeed, researchers have observed a variety of interactions between alcohol and the brain endocannabinoid system, suggesting involvement of this system in alcohol dependence (Colombo et al. 2007). Research on alcohol interactions with the brain endocannabinoid signaling system still is in the early stages. Chronic alcohol has been shown to increase AEA and 2-AG levels in cells and tissue (Basavarajappa and Hungund 1999; Basavarajappa et al. 2000, 2003). There also is evidence that chronic alcohol exposure down-regulates CB1 receptors (Basavarajappa et al. 1998). Clearly, further studies are needed to explore the mechanisms through which endocannabinoids participate in the neural actions of alcohol.

Agonists for the CB1 receptor already are in use, mainly for treating chemotherapy side effects. These agonists reduce nausea induced by chemotherapy and also stimulate appetite in both chemotherapy patients and people with AIDS (Pertwee 2005). Formulations of cannabis plant extracts containing THC and other cannabinoids also have been touted for treatment of multiple sclerosis and other neurological disorders (Wade et al. 2003).

Targeting enzymes involved in endocannabinoid synthesis and degradation also is a promising avenue for future applied pharmacological research (Pertwee 2005). For example, inhibiting the fatty acid amide hydrolase (FAAH) enzyme that metabolizes AEA prolongs the pain-reducing function of the endocannabinoid system in certain paradigms (Hohmann et al. 2005), and strategies like this may be useful in treating certain neuropathic pain disorders.

## Alcohol Interactions With Neurotrophins and Steroid Hormones

Alcohol has been shown to alter intracellular signaling induced by different neurotrophins. The most consistent finding is that acute exposure to alcohol inhibits the signaling and cellular effects produced by BDNF (Davis et al. 1999; Fattori et al. 2008; Li et al. 2004; Ohrtman et al. 2006). This inhibition may contribute to the damaging effects of fetal alcohol exposure on brain development but also appears to affect neurotrophin actions in the adult animal. Studies of chronic alcohol effects on the expression and actions of this neurotrophin are more mixed (reviewed in Davis 2008). Chronic alcohol exposure in adult animals reportedly increases levels of BDNF mRNA in several brain regions (Baek et al. 1996; McGough et al. 2004; Tapia-Arancibia et al. 2001); in a few cases, levels of the protein itself were increased (Bruns and Miller 2007; Miller 2004; Miller and Mooney 2004). However, a number of other studies have reported decreases in BDNF mRNA or no effect (Bowers et al. 2006; MacLennan et al. 1995; Miller et al. 2002; Pandey et al. 1999; Zhang et al. 2000). Some intracellular signaling targets downstream from the Trk receptors, most notably the Fyn and Src protein kinases, also may be targets of acute and chronic alcohol actions (Miyakawa et al. 1997; Wang et al. 2007).

Alcohol exposure has well-known effects on the levels of the corticosteroid hormones released from the adrenal glands. Acute and chronic alcohol exposure enhances levels of cortisol (the major adrenocorticoid hormone in humans), at least in part by increasing secretion of adrenocorticotropic hormone from the pituitary gland (Gianoulakis 1998; Rivier 1996). The hypothalamic–pituitary–adrenal (HPA) axis responsible for brain stimulation of cortisol secretion also is activated during alcohol withdrawal and may be part of a stress-like effect of withdrawal (Adinoff et al. 1998). Within the brain, alcohol also appears to interact with the so-called neurosteroids, including the progesterone metabolites that can potentiate GABA_A_ receptor function. Acute alcohol exposure enhances levels of progesterone in the blood plasma and also increases brain levels of allopregnanolone and other neurosteroids (VanDoren et al. 2000), and there is evidence that this mechanism may contribute to some neurophysiological and behavioral effects of the drug (Biggio et al. 2007; Morrow et al. 2001). All of these steroid compounds are released in response to stressful stimuli, and thus these substances may contribute to interactions between stressful environmental stimuli and the neural actions of alcohol.

## Summary

Communication within the brain involves electrical activation of neurons and chemical transmission between neurons at structures called synapses. Transmission can be broken down into fast synaptic transmission mediated by LGICs and slower-developing neuromodulation mediated by GPCRs. Neurotrophins and steroid hormones also influence neuronal function by altering intracellular signaling pathways and gene expression. Extensive research has shown that many aspects of synaptic transmission are altered by alcohol at doses and brain concentrations encountered during drug ingestion. Alcohol can affect numerous neurotransmitter, neurotrophin, and steroid hormone systems in the brain to produce acute intoxication, as well as neuroadaptations that contribute to tolerance and dependence. The neurotoxic effects produced by alcohol ingestion also involve neurotransmitters. Many of the neurotransmitter systems that are implicated in alcohol actions are currently considered as potential targets for pharmacotherapeutic approaches to combating alcohol abuse and alcoholism. Articles in this two-part series will describe the specific neural effects that contribute to different alcohol actions on the nervous system. Neurotransmitters and synaptic communication, as well as neurotrophins and steroid hormones, figure prominently in these neural effects of alcohol.

## Figures and Tables

**Figure 3A:**
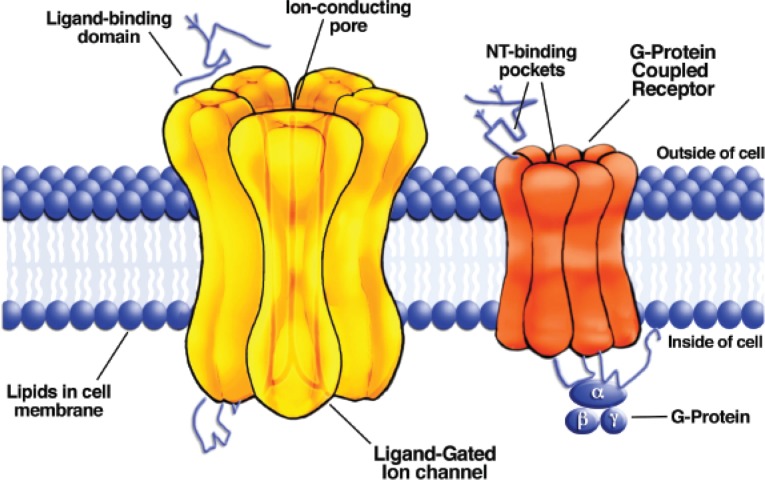
Schematic drawing of a ligand-gated ion channel (left) showing the confluence of individual subunit proteins that define a pore where the ions flow across the cell membrane. A neurotransmitter binds to part of the protein located outside of the cell. Schematic drawing of a G-protein– coupled receptor (right). Neurotransmitter binds either to sites outside the cell or in a “pocket” formed by protein domains that span the membrane. The G-protein that consists of three separate protein subunits (α, β and γ, light blue) is associated with part of the protein inside the cell.

**Figure 3B:**
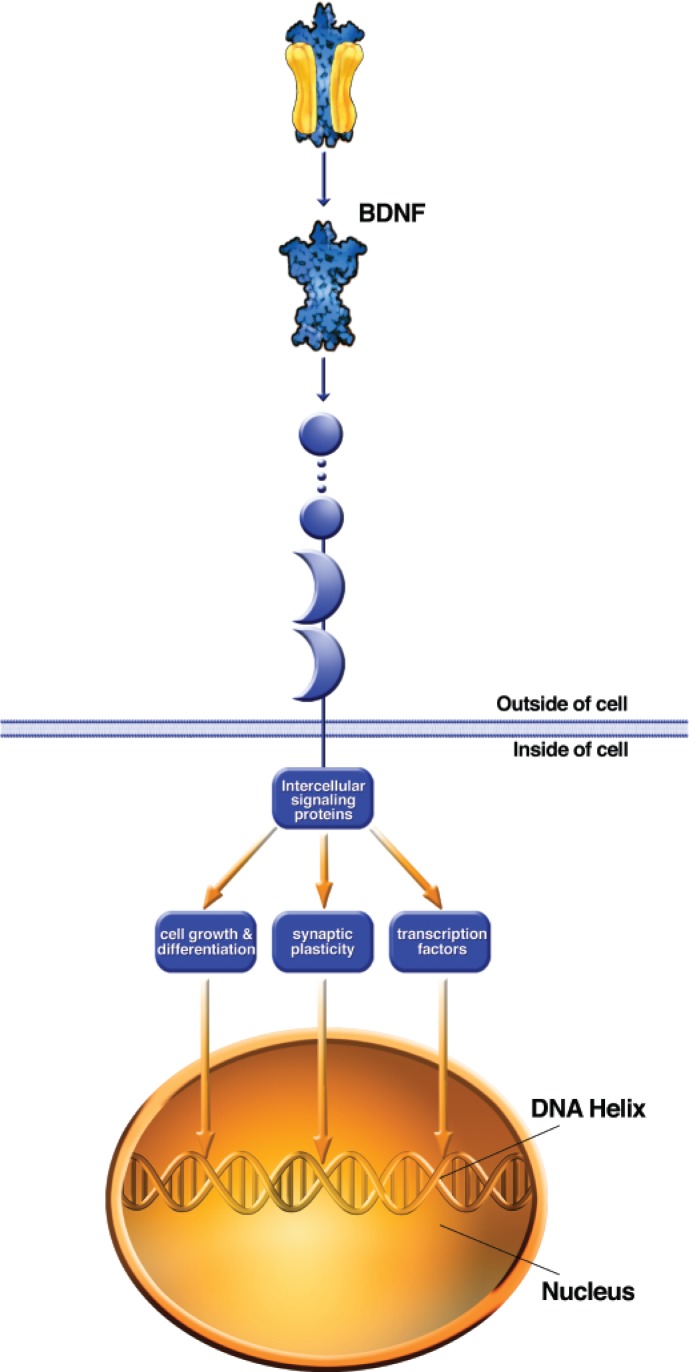
Neurotrophin binding to TRK receptors attracts a variety of intracellular signaling proteins to the intracellular portion of the TrK protein. Activation of these signaling proteins in turn activates transcription factor proteins that act on the nucleus to alter gene expression, as well as other intracellular signaling pathways that promote the growth and differentiation of neurons. Activation of neurotrophin–TrK–intracellular signaling pathways also promotes long-lasting plasticity of synaptic transmission. BDNF = brain-derived neurotrophic factor.

**Figure 3C:**
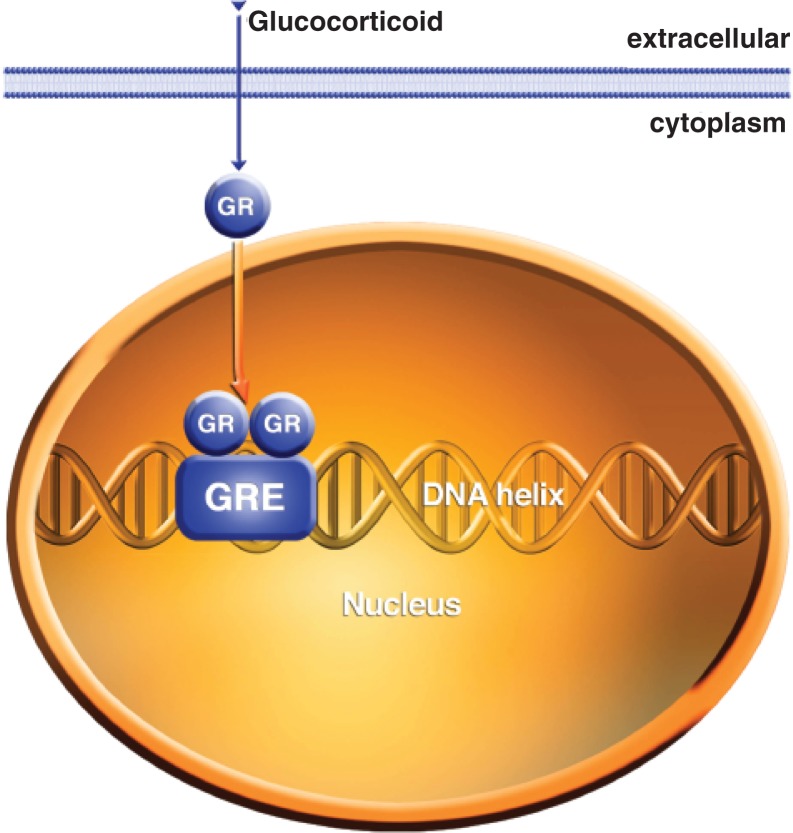
Steroid hormones such as glucocorticoids bind to proteins within the cytoplasm of the cell. Upon steroid binding the protein moves to the nucleus where it can affect protein synthesis at the transcription step. GR = Glucocorticoid receptor; GRE = glucocorticoid response element, a stretch of DNA that binds the GR and activates gene transcription.

## Footnotes

1 For a definition of this and other technical terms, see the Glossary, pp. 279–283.

2 Paracrine means that the substances are released from a given cell and produce their actions only on nearby cells (as opposed to being carried through the bloodstream to distant targets).

3 AMPA (α-amino-3-hydroxy-5-methyl-4-isoxazolepropi-onic acid) is a specific agonist for the AMPA receptor.

4 NMDA (*N*-methyl-d-aspartic acid) is a specific agonist at the NMDA receptor and therefore mimics the action of glutamate on that receptor. In contrast to glutamate, NMDA binds to and regulates the above receptor only but not other glutamate receptors.

5 A second messenger system is a method of cellular signaling, whereby a diffusible signaling molecule is rapidly generated/released which can then go on to activate effector proteins within the cell to exert a cellular response.
